# Identification and antibiogram pattern of *Bacillus cereus* from the milk and milk products in and around Jammu region

**DOI:** 10.14202/vetworld.2018.186-191

**Published:** 2018-02-14

**Authors:** Umar Yusuf, S. K. Kotwal, Sanjolly Gupta, Touqeer Ahmed

**Affiliations:** 1 Division of Veterinary Public Health and Epidemiology, F.V.Sc. & A.H, SKUAST-J, Jammu, Jammu and Kashmir, India; 2 Division of Animal Reproduction Gynaecology and Obstetrics, F.V.Sc. & A.H, SKUAST-K, Srinagar, Jammu and Kashmir, India

**Keywords:** Antibiogram, *Bacillus cereus*, milk, milk products, prevalence

## Abstract

**Aim:**

The aims of the present study were to assess the prevalence, identification, and antibiogram pattern of *Bacillus cereus* from 215 samples of different milk and milk products in and around Jammu region.

**Materials and Methods:**

In the present study, 215 samples of milk, rasgulla, burfi, rasmalai, kalaari, paneer, ice cream, and pastry were collected and analyzed for the isolation of the *B. cereus* using PEMBA, and antibiogram pattern was observed for all the milk and milk products.

**Results:**

*B. cereus* was detected in 61/215 samples with an overall prevalence of 28.37%. Biotyping revealed predominantly 5, 7, and 2 biotypes in raw milk. Burfi and ice cream revealed 2, 3, 5, and 7 biotypes. Rasgulla had 2, 3, and 5 biotypes; paneer and rasmalai had biotypes 2 and 5, while kalaari revealed biotype 5. Antibiogram pattern revealed that isolates were highly sensitive to gentamicin (100%), intermediate to ampicillin (40.98%), tetracycline (31.14%), erythromycin (29.50%), and amoxicillin (26.22%), and high resistance against penicillin G (100%). Adulteration of starch was detected in 16.66 % raw milk samples. All starch positive samples were positive for *B*. *cereus*. However, 12 starch negative samples also yielded *B*. *cereus*.

**Conclusion:**

From this study, it was concluded that highest prevalence of *B*. *cereus* was found in ice cream. Several isolates of *B. cereus* showed toxigenic activity, so the presence of *B. cereus* in milk and milk products may be of public health hazard. The antibiogram pattern of *B. cereus* isolates showed sensitivity to gentamicin, ciprofloxacin, chloramphenicol, streptomycin, and resistance to penicillin-G and cephalexin. The presence of *B. cereus* in milk and milk products showed a strong association besides establishing the fact that starch adulteration can be indicative of the presence of *B. cereus*.

## Introduction

*Bacillus cereus* is a Gram-positive, motile, spore-forming, rod-shaped bacterium. *B. cereus* is associated with two distinct types of gastrointestinal disorders, the diarrheal and emetic syndrome [1,2]. There are different species of *Bacillus*, of which the *B. cereus* is the most important microorganism involved in the spoilage of dairy environment [3]. In general, *B. cereus* is sensitive to gentamicin, chloramphenicol, vancomycin, and ciprofloxacin, while highly resistant to penicillin G [4-7].

*B. cereus* is of concern because of its nature and growth in the dairy environment [8]. The isolates of *B. cereus* from products of dairy origin produce lipase, and other proteases which are thermostable, i.e., have the ability to remain active after pasteurization temperature, which leads to the defects in the final dairy products [3,9]. Since it has the ability to produce toxins and enterotoxins, it poses a concern for the dairy industry as it causes food poisoning. The adherence property of *B. cereus* to the dairy plant stainless steel surfaces poses a risk to health and serious hygiene issues. There is the loss of both product and equipment due to biofuel production, hence leading to economic loss due to spoilage [8]. Furthermore, the spore-forming and psychotropic nature of certain *B. cereus* strains ensure the survival and multiplication of bacteria during milk processing. As a result, the dairy industry often reported food poisoning outbreaks due to this organism [8].

India being the largest producer of milk in the world has high per capita milk consumption and also of milk products. The same stands true for Jammu and Kashmir. However, the milk production and processing practices are of low standards and may lead to contaminated milk and milk products which in turn can predispose people to milk-borne infectious agents including *B. cereus*. Keeping in view the above facts, the objective was framed to study the prevalence, identification, and antibiogram of *B. cereus* from milk and milk products in and around Jammu region.

## Materials and Methods

### Ethical approval

No ethical approval was required for the study because no live animal was involved in the study. The samples of different milk and milk products were collected in and around Jammu region.

### Media used

In the current study, polymyxin pyruvate egg yolk mannitol bromothymol blue agar (PEMBA), *B. cereus* agar base, brain heart infusion broth (BHIB), Muller-Hinton agar, sheep blood agar, nitrate broth, motility test medium, and Simmons citrate agar were used.

### Bacterial strain

Reference strain of *B. cereus* (MTCC 1272) obtained from the microbial type culture collection and gene bank (MTCC), Institute of Microbial Technology (IMTECH), Chandigarh, India, was used for standardization of techniques. The strain was maintained by periodic subculturing on a nutrient agar slant.

### Sampling

There were a total of 215 samples, of which 30 samples of raw milk were collected in sterile containers from local milk vendors, and 185 samples of milk products comprised of 29 burfi, 28 rasgulla, 28 rasmalai, 25 ice cream, 25 paneer, 25 pastry, and 25 kalaari were collected aseptically from various retail outlets in and around Jammu city. The samples were brought to the laboratory on ice and processed within 2 h of collection.

### Isolation and identification of *B. cereus*

#### Isolation

PEMBA media were used for isolation of *B. cereus*. The presumed isolates were purified on nutrient agar slants for further characterization. All presumptive colonies of *B. cereus* were purified and subjected to morphological and biochemical tests for identification and confirmation as described in Bacteriological Analytical Manual of United States Food and Drug Administration [10] and Bergey’s Manual of Systematic Bacteriology [11].

### Biotyping

The isolates were biotyped on the basis of their ability to ferment ammonium salt sugars (ASS), namely, xylose, salicin, and cellobiose as per the scheme proposed by Jha and Narayan [12] (Table-1).

**Table-1:** Biotyping scheme as proposed by Jha and Narayan [12].

Biotype	Xylose	Salicin	Cellobiose
1	-	-	-
2	+	-	+
3	-	+	-
4	+	-	-
5	+	+	+
6	-	-	+
7	-	+	+
8	+	+	-

### Antibiogram pattern of *B. cereus*

All the *B*. *cereus* isolates were examined for their antibiogram pattern by disc diffusion technique as described by Bauer *et al*. [13] against a panel of 10 antibiotics. The results were interpreted as sensitive or resistant based on CLSI interpretive standards HiMedia [14].

### Detection of starch/rice flour adulteration [15]

The samples of milk and milk products were subjected to test for detection of starch/rice flour as an adulterant. 3 ml of the test sample (raw milk/milk ­product) was thoroughly mixed and boiled for 15 min. After cooling to the room temperature, 2-3 drops of 1 % iodine was added drop by drop. A positive test was indicated by the appearance of blue color, which disappeared after boiling and reappeared on cooling.

## Results

The present study was carried out to explore the prevalence of *B. cereus* in milk and milk products collected from Jammu city. *B. cereus* was isolated and identified on the basis of cultural, morphological, and biochemical characters.

### Isolation and identification of *B. cereus*

On PEMBA medium, typical fimbriate peacock blue-colored colonies (3-5 mm) surrounded by a blue zone of egg yolk hydrolysis against green/greenish-yellow background was presumed as *B. cereus*. The presumed isolates were purified on nutrient agar slants and were subjected to standard biochemical and other tests for further characterization. After enrichment of each sample in BHIB, inoculums were streaked on PEMBA medium.

All the isolates were identified morphologically and biochemically. All the isolates were Gram-positive, large rods, and sometimes, spores were also observed. Motility test of *B. cereus* was performed in 18-h-old BHIB which showed characteristic swarming and swimming motility in hanging drop under 40× cavity slide. All the isolates were positive for catalase test, citrate utilization test, Voges–Proskauer test, and nitrate reduction test but negative for oxidase test. The isolates also hydrolyzed starch and produced hemolysis on sheep blood agar. All the isolates produced acid from glucose and mannose but failed to produce acid from mannitol and arabinose.

61 samples were found positive for *B. cereus* with an overall prevalence of 28.37%. The present study revealed maximum contamination of ice cream, rasgulla, and burfi with *B. cereus*, followed by pastry, raw milk, rasmalai, paneer, and kalaari (Table-2)

**Table-2:** Prevalence of *B. cereus* in raw milk and milk products.

Milk product	Samples tested	Samples positive	% prevalence
Raw milk	30	8	26.66
Burfi	29	10	34.48
Rasgulla	28	11	39.28
Rasmalai	28	7	25
Ice cream	25	11	44
Paneer	25	4	16
Pastry	25	8	32
Kalaari	25	2	8
Total	215	61	28.37

*B. cereus=Bacillus cereus*.

### Biotyping

The isolates of *B. cereus* were biotyped as per the scheme proposed by Jha and Narayan [12] by demonstrating their fermentation reaction in ASS, namely, xylose, salicin, and cellobiose. Overall predominant biotypes recovered were biotypes 5 followed by 2, 7, and 3. From rasgulla, biotypes 2, 3, and 5 were recovered. Burfi and ice cream revealed 2, 3, 5, and 7 biotypes, while pastry revealed biotypes 2, 5, and 7. Paneer and rasmalai revealed biotypes 2 and 5 only. In Kalaari, only biotype 5 was present (Table-3).

**Table-3:** Results of biotyping of *B. cereus* isolates.

Samples	Biotype 2	Biotype 3	Biotype 5	Biotype 7
Raw milk	2	-	3	3
Burfi	3	2	4	1
Ice cream	3	2	3	3
Rasmalai	2	-	5	-
Rasgulla	6	1	4	-
Paneer	2	-	2	-
Pastry	2	-	4	2
Kalaari	-	-	2	-
Total	20	5	27	9

*B. cereus=Bacillus cereus*.

### Antibiogram pattern of *B. cereus*

Based on CLSI interpretive standards for Gram-positive and/or aerobic bacteria, the antibiotics to which *B. cereus* isolates were susceptible included gentamicin, ciprofloxacin, and chloramphenicol followed by streptomycin. On the contrary, all isolates depicted high resistance to penicillin G and cephalexin. Less than 50% resistance was recorded for ampicillin, tetracycline, erythromycin, and amoxicillin (Table-4).

**Table-4:** Antibiogram resistance pattern of *B. cereus* isolates.

Samples (n)	S (%)	P (%)	C (%)	G (%)	T (%)	Am (%)	CN (%)	E (%)	A (%)	Cip (%)
Milk (8)	1 (12.5)	8 (100)	1 (12.5)	0	2 (25)	1 (12.5)	5 (62.50)	2 (25)	3 (37.5)	0 (0)
Ice cream (11)	2 (18.18)	11 (100)	1 (9.09)	0	5 (45.45)	3 (27.27)	11 (100)	4 (36.36)	5 (45.45)	0 (0)
Burfi (10)	2 (22.22)	10 (100)	0	0	2 (22.22)	2 (22.22)	9 (100)	3 (33.33)	4 (44.44)	1 (10)
Rasgulla (11)	1 (10)	11 (100)	0 (0)	0	3 (30)	3 (30)	10 (100)	2 (20)	4 (40)	0 (0)
Pastry (8)	2 (25)	8 (100)	2 (25)	0	3 (37.5)	2 (25)	8 (100)	2 (25)	4 (50)	1 (12.5)
Paneer (4)	0	4 (100)	0 (0)	0	1 (25)	1 (25)	3 (75)	1 (25)	1 (25)	0 (0)
Rasmalai (7)	1 (14.28)	7 (100)	1 (14.2)	0	2 (28.57)	2 (28.57)	7 (100)	3 (42.8)	4 (57.14)	0 (0)
Kalaari (2)	0	2 (100)	0 (0)	0	1 (50)	2 (100)	2 (100)	1 (50)	0 (0)	0 (0)
Total (61)	9 (14.75)	61 (100)	5 (8.19)	0 (0)	19 (31.14)	16 (26.22)	55 (90.16)	18 (29.50)	25 (40.98)	2 (3.27)

n=Number of isolates, S=Streptomycin, P=Penicillin G, C=Chloramphenicol, G=Gentamicin, T=Tetracycline, Am=Amoxicillin, CN=Cephalexin, E=Erythromycin, A=Ampicillin, Cip=Ciprofloxacin, *B. cereus=Bacillus cereus*

### Antibiogram pattern of *B. cereus* isolates from milk and different milk products

Of eight isolates, two isolates showed intermediate sensitivity to streptomycin and erythromycin, while one isolate showed intermediate sensitivity to tetracycline, amoxicillin, cephalexin, and ampicillin. All the isolates were sensitive to gentamicin and ciprofloxacin while being resistant to penicillin G.

### Antibiogram pattern of *B. cereus* isolates from ice cream

Of eleven isolates, three isolates showed intermediate sensitivity to streptomycin and amoxicillin, and two isolates showed intermediate sensitivity to erythromycin and ampicillin. Five isolates were resistant to tetracycline and ampicillin, while ten isolates were susceptible to chloramphenicol and ciprofloxacin. All the isolates were sensitive to gentamicin but resistant to penicillin G.

### Antibiogram pattern of *B. cereus* isolates from pastry

Of eight isolates, two isolates showed intermediate sensitivity to ampicillin and streptomycin and one isolate to erythromycin. Two isolates showed resistance to streptomycin, chloramphenicol, amoxicillin, and erythromycin. All isolates were susceptible to gentamicin while resistant to cephalexin and penicillin G.

### Antibiogram pattern of *B. cereus* isolates from burfi

Of ten isolates, two isolates showed resistance to streptomycin, tetracycline, and amoxicillin. Three isolates showed resistance to erythromycin and four isolates showed resistance to ampicillin. All the isolates were susceptible to ciprofloxacin, gentamicin, and chloramphenicol. Intermediate sensitivity was shown by two isolates to streptomycin, three isolates to amoxicillin, and one isolate to ampicillin.

### Antibiogram pattern of *B. cereus* isolates from rasmalai

All seven isolates were resistant to penicillin G and cephalexin while susceptible to gentamicin and ciprofloxacin. Only two isolates showed intermediate sensitivity to streptomycin and amoxicillin. One isolate showed resistance to streptomycin and chloramphenicol, and two isolates showed resistance to tetracycline and amoxicillin. Three and four isolates showed resistance to erythromycin and ampicillin, respectively.

### Antibiogram pattern of *B. cereus* isolates from rasgulla

All eleven isolates were susceptible to ciprofloxacin, gentamicin, and chloramphenicol while resistant to penicillin G. Three isolates showed intermediate sensitivity to streptomycin and erythromycin while resistance to tetracycline and amoxicillin.

### Antibiogram pattern of *B. cereus* isolates from Kalaari

Of two isolates, one isolate showed resistance to tetracycline and erythromycin. All the isolates were resistant to penicillin G, amoxicillin, and cephalexin while susceptible to streptomycin, gentamicin, chloramphenicol, ampicillin, and ciprofloxacin.

### Antibiogram pattern of *B. cereus* isolates from paneer

Of four isolates, two isolates showed intermediate sensitivity to ampicillin and one isolate showed intermediate sensitivity to amoxicillin. One isolate showed resistance to tetracycline, amoxicillin, erythromycin, and ampicillin. All the isolates were susceptible to chloramphenicol, streptomycin, gentamicin, and ciprofloxacin.

### Starch adulteration in raw milk and milk products

Of 215 samples of raw milk and milk products, 49 samples were found adulterated with the starch (rice flour) showing an overall adulteration of 22.79%.

Maximum starch adulteration among milk and milk products was observed in rasgulla with 10/28 (35.71%) and burfi with 10/29 (34.48%) samples positive followed by pastry and ice cream, 8/25 (32.00%), rasmalai 5/28 (17.85%), and raw milk 5/30 (16.66%). Paneer and kalaari showed the least adulteration with only 2 (8%) and 1 (4%) sample positive, respectively (Table-5).

**Table-5:** Prevalence of starch adulteration in raw milk and milk products.

Sample	Samples tested	Samples adulterated	Total %
Raw milk	30	5	16.66
Burfi	29	10	34.48
Rasgulla	28	10	35.71
Rasmalai	28	5	17.85
Ice cream	25	8	32
Pastry	25	8	32
Paneer	25	2	8
Kalaari	25	1	4
Total	215	49	22.79

## Discussion

Milk and milk products may serve as potential reservoirs for many bacterial pathogens. In the present study, all the 215 samples of milk and milk products were subjected to standard microbiological procedures for the detection of *B. cereus*. The study revealed 26.66% of raw milk samples contaminated with *B. cereus*. Sharma *et al*. [16] reported 66% contamination of milk with *B. cereus in* Ludhiana which was lower than the results of the current study.

In milk products, the highest prevalence of *B. cereus* was observed in ice cream (44%), followed by rasgulla (39.28%), burfi (34.48%), pastry (32%), and rasmali (25%). Least prevalence was observed in paneer (16%) and kalaari (8%) samples. These results are in agreement with Hassan *et al*. [17] who reported a prevalence of 30%, 48%, and 2% in raw milk, ice cream, and yogurt, respectively, in Egypt. In Ludhiana, Sharma *et al*. [16] reported 44.8 and 44.4% incidence in burfi and milk powder, respectively.

Contamination of milk and milk products by *B. cereus* has been reported by many workers and established that entry of *B. cereus* in the raw milk and milk products could be due to many reasons including the adulteration of milk by water and rice flour [18] and use of contaminated utensils [19]. Another possible explanation could be the prolonged holding time under unhygienic conditions at retail outlets, resulting in a higher initial load of *B. cereus* in raw milk. Recovery of *B. cereus* from pasteurized milk has also been reported.

The morphological and biochemical characteristics of *B. cereus* isolates in the present study closely resembled those exhibited by the standard strain. It was interesting to note that majority of the isolates from milk and milk products differed in their response to the fermentation reaction to xylose, salicin, and cellobiose, on the basis of a biotyping scheme for *B. cereus* as proposed by Jha and Narayan [12]. This scheme was used exclusively for locating the source of ­contamination of different foods and differentiation of various strains [20,21]; however, to make the scheme more reliable and expressive, the higher number of samples from other possible sources needs to be screened.

In the present study, the biotyping pattern of isolates from the raw milk revealed predominance of biotypes 5, 7, and 2 with 37.5% isolates typed as biotypes 5 and 7 and 25% as biotype 2. Burfi and ice cream revealed 2, 3, 5, and 7 biotypes. Rasgulla harbored biotypes 2, 3, and 5; paneer and rasmalai were contaminated with biotypes 2 and 5, while kalaari revealed biotype 5 only. Previously, Yadav [20] reported a predominance of biotypes 7, 6, and 5 from fried rice and boiled rice; Willayat [21] reported biotypes 7, 6, 5, and 1. Javed [22] reported the predominance of biotypes 1, 7, 5, and 2 in boiled rice and 3, 4, and 2 in the raw milk. In another study [23] in Kashmir region, the presence of biotypes 3, 4, 1, and 6 were reported from the mutton tikka samples and biotypes 6, 7, 5, and 2 from the chutneys. It is documented that biotypes 2, 5, and 7 are frequently associated with starchy foods and the preponderance of these biotypes in milk and milk products in the present study may indicate the adulteration of these products with starchy products.

Keeping in view the above facts, the antibiotic sensitivity of all the *B*. *cereus* isolates against a panel of ten commonly used antibiotics was carried out. The antibiotics to which most of the isolates were susceptible included gentamicin (100%), ciprofloxacin (96.73%), and chloramphenicol (91.81%) followed by streptomycin (85.25%). The isolates were resistant to penicillin G (100%) and cephalexin (90.16%). However, <50% resistance was recorded for ampicillin (40.98%), amoxicillin (26.22%), tetracycline (31.14%), and erythromycin (29.50%). These findings are in accordance with the findings of Schlegelova *et al*. [4], Turnbull *et al*. [5], Whong and Kwaga [6], and Kursun *et al*. [7]. Hafeez *et al*. [23] also reported that the strains of *B. cereus* isolated from the vendor samples were highly resistant to penicillin G (92.59%). Previous work on the antimicrobial susceptibility of *B. cereus* revealed higher susceptibility to streptomycin, chloramphenicol, erythromycin, and ciprofloxacin and less susceptible to ampicillin, ampiclox, clotrimazole, and cloxacillin [24]. Variations in the percentages may be due to the differences in the concentrations of antimicrobial agents used, differences in the source of isolates, drug resistance transfer, and overall wide use of the antibiotics. The development of drug resistance against certain antibiotics may be due to the use of these drugs in medical and veterinary practice to treat infections and misuse of the drugs that could have led to drug resistant strains.

Milk and milk products were also subjected to starch adulteration test, and both starch positive as well as starch negative samples were tested for detection of *B. cereus* by standard procedure. The study revealed 16.66% raw milk samples adulterated with starch. Among milk products, rasgulla was found to be highly adulterated (35.71%) followed by burfi (34.48%), ice cream and pastry (32%), rasmalai (17.85%), paneer (8%), and kalaari (4%). The higher % age of adulteration in rasgulla and burfi may possibly be due to use of the starchy substrate in the manufacture of these products. The lesser % age of adulteration in paneer and kalaari could be because of non-feasibility of adulteration keeping in view the texture, taste, and other factors vital for the acceptability of these products.

In the present study, all the starch positive samples of the milk and milk products revealed the presence of *B. cereus* which otherwise form the part of the microflora of the rice and rice products, establishing a fact that rice powder may have been adulterant for increasing the physical attributes, texture, and the bulk of the product. However, the starch negative samples of milk and milk products have also revealed positive results for *B. cereus* and the latter might have entered in the processing line from the water, utensils, or other sources.

The present study unveils the faulty practice of adulteration of milk and milk products with starch, which necessitates stronger monitoring and frequent testing followed by stringent control measures on the part of control authorities. Since all the starch positive samples were confirmed for the presence of *B. cereus*, starch adulteration may act as an indicator for the presence of *B. cereus* in milk and milk products.

## Conclusion

From the study, it was concluded that the overall prevalence of *B. cereus* in milk and milk products was 28.37%. The highest prevalence of *B. cereus* was found in ice cream followed by rasgulla, burfi, pastry, rasmalai, paneer, and kalaari samples. Several isolates of *B*. c*ereus* showed toxigenic activity, so the presence of *B*. *cereus* in milk and milk products may be a public health hazard. The antibiogram pattern of *B. cereus* isolates showed sensitivity to gentamicin, ciprofloxacin, chloramphenicol, and streptomycin and resistance to penicillin-G and cephalexin. Starch adulteration and *B. cereus* contamination in milk and milk products showed a strong association besides establishing the fact that starch adulteration can be indicative of the presence of *B. cereus*. Besides, *B. cereus* may cause non-gastrointestinal tract syndromes such as meningitis, endocarditis, and septicemia particularly in children and the older people. The effective use of quality control and safety measures during production/manufacture besides creating awareness among food handlers and consumers becomes necessary to ward off the menace of *B. cereus* and other food poisoning organisms.

## Authors’ Contributions

This study was carried out as a part of MVSc study of UY, under the guidance of SKK. SG was actively involved in the whole study, and TA has helped in various ways and designed the manuscript for publication. All the authors have read and approved the final manuscript.
